# Are shed hair genomes the most effective noninvasive resource for estimating relationships in the wild?

**DOI:** 10.1002/ece3.6157

**Published:** 2020-05-18

**Authors:** Anubhab Khan, Kaushalkumar Patel, Subhadeep Bhattacharjee, Sudarshan Sharma, Anup N. Chugani, Karthikeyan Sivaraman, Vinayak Hosawad, Yogesh Kumar Sahu, Goddilla V. Reddy, Uma Ramakrishnan

**Affiliations:** 1 National Centre for Biological Sciences, TIFR, Bangalore, India; 2 Rajasthan Forest Department, Jaipur, India; 3 Department of GEMES, University of Johannesburg, Johannesburg, South Africa; 4 Medgenome Labs Pvt. Ltd, Bangalore, India

**Keywords:** genome, noninvasive sample, pedigree, Ranthambore Tiger Reserve, relatedness, relationships, shed hair

## Abstract

1. Knowledge of relationships in wild populations is critical for better understanding mating systems and inbreeding scenarios to inform conservation strategies for endangered species. To delineate pedigrees in wild populations, study genetic connectivity, study genotype-phenotype associations, trace individuals, or track wildlife trade, many identified individuals need to be genotyped at thousands of loci, mostly from noninvasive samples. This requires us to (a) identify the most common noninvasive sample available from identified individuals, (b) assess the ability to acquire genome-wide data from such samples, and (c) evaluate the quality of such genome-wide data, and its ability to reconstruct relationships between animals within a population.
2. We followed identified individuals from a wild endangered tiger population and found that shed hair samples were the most common compared to scat samples, opportunistically found carcasses, and opportunistic invasive samples. We extracted DNA from these samples, prepared whole genome sequencing libraries, and sequenced genomes from these.
3. Whole genome sequencing methods resulted in between 25%–98% of the genome sequenced for five such samples. Exploratory population genetic analyses revealed that these data were free of holistic biases and could recover expected population structure and relatedness. Mitochondrial genomes recovered matrilineages in accordance with long-term monitoring data. Even with just five samples, we were able to uncover the matrilineage for three individuals with unknown ancestry.
4. In summary, we demonstrated that noninvasive shed hair samples yield adequate quality and quantity of DNA in conjunction with sensitive library preparation methods, and provide reliable data from hundreds of thousands of SNPs across the genome. This makes shed hair an ideal noninvasive resource for studying individual-based genetics of elusive endangered species in the wild.

## 1 Introduction

Long-term monitoring of individuals and their relatedness within populations provides key insights into demography, reproductive success, fitness, and social organization (Kruuk & Hill, 2008; Pemberton, 2008). Estimating genetic relatedness in endangered populations is crucial for evaluation of mating patterns and management strategies. Relationships between individuals and genetic relatedness among individuals have been key to understanding communities (Vigilant, Hofreiter, Siedel, & Boesch, 2001; Widdig, Nürnberg, Krawczak, Streich, & Bercovitch, 2001), predict survivability (Bean et al., 2004), and fitness (Frère et al., 2010). Ongoing habitat fragmentation has resulted in small and isolated populations for many carnivores (Crooks, 2002; Haddad et al., 2015), and evaluation of relationships among individuals is becoming an increasingly important part of conservation planning and management, especially for large mammals.

Estimating maternal relationships often requires tracking and following individuals, and monitoring their reproductive success. Molecular genetic data are essential to investigate paternal relationships and cryptic relatedness between individuals (Slate, Marshall, & Pemberton, 2000). This is especially true for elusive species where matings cannot be observed or paternal care is absent. Recent studies have used genome-wide markers to investigate paternity, relatedness, and even population-level pedigrees (Hadfield, 2012; Huisman, 2017; Weir, Anderson, & Hepler, 2006). Typically, such studies require capture of wild individuals, tagging, and blood sample collection (Clutton-Brock & Pemberton, 2004). While this approach is possible for some herbivores, it is difficult to implement for elusive, endangered, large carnivore species. In most cases, immobilization may be logistically challenging or dangerous. For such species, minimally invasive samples like scat matter (Solberg, Bellemain, Drageset, Tab erlet, & Swenson, 2006), excreted waste, pellets, saliva swabs from kill sites, environmental DNA or samples of shed skin, feather (Horváth, Martínez-Cruz, Negro, Kalmár, & Godoy,2005), antler, and hair are more feasible (Rozhnov et al., 2009). However, most of these samples yield low quantities of DNA (Ball et al., 2007; Gupta, Kumar, & Hussain, 2013). Such noninvasive samples have varying percentages of host DNA depending on the sample source. For example, DNA from scat samples is dominated by bacterial DNA (Chiou & Bergey, 2018) and prey DNA, while urine samples (if not already mixed with environmental DNA from soil or surface) have low amount of DNA and high rate of allelic dropout in microsatellite data (Caragiulo et al., 2015). Saliva samples from kill sites may belong to more than one individual, and have bacterial and prey DNA contamination. Shed hair samples are expected to be enriched in host DNA but are potentially scarce at a site. Hair samples have been used to sequence and assemble whole genomes of extinct woolly mammoths (Miller et al., 2008).

Here, we attempt to identify the most common noninvasive sample sources in the field from identified individuals and the potential of these samples for recovering relatedness among individuals in wild populations. In order to do so, we sampled shed hair, scat, opportunistically found carcasses and blood from individuals in a wild tiger population. Tigers are elusive and endangered large felids, making it difficult to sample them invasively. Because tigers have unique stripes, it is possible to identify individuals visually. First, we investigated the most frequently encountered samples from identified individuals and tested whether these samples (a) can be collected in enough amounts for genome sequencing in pragmatic amounts of time and (b) yield more genome-wide information than other noninvasive or invasive samples in the context of identified individuals. Finally, we assessed whether the genome-wide data generated provide biologically meaningful insights by investigating (a) documented/known patterns of population structure and (b) cases of known and unknown maternity and relatedness.

## 2 Materials and Methods

### 2.1 Ethical statement

Samples were collected in collaboration with the forest department as per their guidelines. Permission was granted in letter number 19 (Part-Uma) Permission/Research/CWLW/2017 dated 15/12/2017.

### 2.2 Zoo and field sampling

We collected samples from a wild-caught tiger named T24 housed in a lone enclosure in a zoo to optimize DNA extraction and sequencing. Specifically, we collected shed hair in scratch marks on trees and on the ground where the tiger had been resting. In the wild (in Ranthambore Tiger Reserve), we conducted sampling as depicted in Box 1. Briefly, we obtained information about ranging patterns of tigers from forest officials and searched for individuals in their known territories. We followed 34 wild tigers over 255 days total during three field seasons, from 20 May to 30 June 2017, 1 to 30 November 2017, and 1 January to 31 June 2018. After locating an individual, we followed it in a vehicle. If an individual scratched a tree or bush or rested in a spot, we waited for the individual to leave and then collected hair from these areas using clean forceps. This was repeated until an individual was sampled a minimum of 5 times. We collected scat samples from individuals by swabbing the surface of the scat using a sterile swab dipped in Longmire’s buffer (Longmire et al., 1997) and preserved it in Longmire’s buffer until further processing in the laboratory. Both the sample types were transferred to a −20°C freezer within 48 hr of collection.

We collected tissue from the carcass of one individual, named T16, in absolute ethanol and transported to the laboratory in gel packs. We also collected blood from one tranquilized individual, named T104, in PAXgene Blood DNA Tube (Cat. 761115) and transported to the laboratory in dry ice.

### 2.3 Laboratory methods

To test the relative effectiveness of sample type as a source of whole genome DNA, we used hair samples from five tigers: T24, T20, T47, T64, and T104; tissue from two tigers (T16 and T104); and scat samples from three tigers (T03, T08, and T47; Table S1).

#### 2.3.1 DNA extraction

We first tested if DNA from hair root or whole hair is better suited for whole genome sequencing. For this, we used samples from T24 and T47. We extracted DNA using the approaches depicted in Box 2. Briefly, for the hair root only method, we selected 10 hair roots from the zoo individual and discarded the hair shaft. To these, we added 200 μl of AL buffer, 40μl of Proteinase K, and 20 μl of 1 M DTT and incubated overnight at 56°C. These hair roots were extracted using a modified protocol of the Qiagen blood and tissue extraction kit (Cat. 69504). DNA from hair root was extracted for tigers T24 and T47 only.

For the whole hair DNA extraction, we randomly selected 8–14 hair strands (irrespective of presence of a visible hair root) for an individual from a sampling site and rinsed in 0.1X commercial bleach and washed with nuclease-free water in order to remove any DNA on the surface. To this, we added 300 μl ATL buffer (Qiagen), 30 μl Proteinase K, and 20 μl 1M DTT and incubated at 56°C until visible lysis (noted by visible reduction to disappearance of hair volume). To this lysate, we added 300 μl AL buffer (QIAGEN), 3 μl of 1 μg/μl carrier RNA, and 300 μl of absolute alcohol (in that order); vortexed; and loaded onto the spin column. The rest remains the same as mentioned in Qiagen blood and tissue extraction kit handbook. DNA from whole hair was extracted for tigers T20, T24, T47, T64, and T104.

We extracted DNA from tissue samples of tigers T16 and T104 using Qiagen blood and tissue extraction kit (Cat. 69504) as per the manufacturer’s instructions. Scat samples from T47, T08, and T03 were extracted using the method described in Natesh et al. (2019).

#### 2.3.2 Library preparation and Sequencing

We prepared DNA whole genome libraries using NEBNext^®^ UltraTM II DNA Library Prep Kit (Cat. E7645L, NEB Inc). DNA was quantified on Qubit™ 3.0 fluorometer using Qubit High sensitivity dsDNA Assay (#Q32854, Thermo Fisher Scientific). Quantified DNA was then fragmented by sonication using Covaris LE220 ultrasonicator using Covaris microTUBE (#520053, Covaris^®^ Inc) to obtain a final insert size of 250–350 bp. Next, the DNA fragments were taken for end repair step where blunt ends are created on either side of the fragments. A single “A” nucleotide was added on the 3′ ends of the fragments to facilitate the ligation of NEB stem-loop adapters. Ligated products were then cleaned, and size selected using Agencourt AMPure XP beads (#A63882, Beckman Coulter). These size selected products were amplified using limited cycle PCR. Twelve cycles for whole hair and hair follicle and eight cycles for tissues, during which indices (Barcodes) and flow-cell binding sequences, were added. After a final cleanup with Agencourt AMPure XP beads, the libraries were quantified using Qubit DNA Assay and the fragments were assessed using DNA TapeStation D1000 Screen Tape (#5067-5582,5583, Agilent Technologies). The quantified libraries were then clonally amplified on a cBOT and sequenced on the HiSeq X with 150bp paired end chemistry.

### 2.4 Analyses

#### 2.4.1 Data processing

We trimmed the reads from 150 bp paired end sequencing using TRIMMOMATIC (Bolger, Lohse, & Usadel, 2014) to have an mean PHRED scaled quality of 30 in a sliding window of 15 bp, and any read that was shorter than 36 bp after trimming was removed from further analysis. We aligned these reads to (a) the tiger genome assembly (Armstrong et al., 2019) and (b) the mitochondrial genome of tiger (NC_010642.1) using BOWTIE2 (Langmead & Salzberg, 2012). We then saved the alignments in a binary format using SAMTOOLS1.9 (Li et al., 2009). We marked duplicated trimmed reads using the MarkDuplicates option in PICARD (http://broadinstitute.github.io/picard). To assess the quality of the alignments, QUALIMAP (García-Alcalde et al., 2012) was used. We noted the percent mapped reads, percent duplicate reads, and percent genome covered at least 1X depth.

#### 2.4.2 Sample dependent data quality

To test for difference in data quality across different kinds of samples, we used data from whole genomes from the muscle and blood tissue (for tigers T16 and T104), shed hair (for tiger T24, T20, T64, T47, and T104), and scat extracts (for tigers T03, T08, and T47). We subsampled data from samples with a higher number of reads to match the samples with the lowest number of reads (i.e., 132,602,774 reads). Comparison of the raw data without controlling for number of reads is presented in Table S2.

To test for differences in sequences obtained due to use of different sample types, we estimated the percent of loci that mismatched other samples of the same individual. For this, we repeat masked the reference genome as described in Armstrong et al. (2019) where we used the Felidae database in RepeatMasker (http://www.repeatmasker.org) to identify known repeats in the genome. We then called variants for the entire dataset using bcftools multiallelic caller (Li et al., 2009). We adjusted the mapping quality of the reads during mpileup using -C50. The raw variants were filtered for Quality and Genotype quality of 30 (this ensured we have 99.9% confidence in the bases and the genotypes) and depth of 10 and removed indels (this ensured only SNPs were being used). We estimated mismatches (0: identical; 1: single allele mismatch; and 2: both alleles mismatch) between scat, hair root, and whole hair genome SNPs for tiger T47. In one other case, we compared SNPs called from whole hair and blood from tiger T104. We subsampled the variant (vcf) files to contain only the samples being compared (Table S3). For scat versus hair root genome and scat versus whole hair genome, we obtained 12,583 and 20,760 SNP loci, respectively, with no missing data. For hair root genome versus whole hair genome, we obtained 34,601 SNP loci, and for whole hair genome versus blood genome we obtained 62,765 SNP loci with no missing data. On these files, we used the genome function of PLINK (Purcell et al., 2007) to obtain the pairwise mismatches.

For the whole dataset, we called variants using the best quality samples using bcftools multiallelic caller (Li et al., 2009). We adjusted the mapping quality of the reads during mpileup using -C50. The raw variants were filtered for Quality and Genotype quality of 30, depth of 10, maximum missing data allowed per locus of 20%, conformity of Hardy–Weinberg equilibrium at a *p*-value of .05, and minor allele count of 3, with no indels.

#### 2.4.3 Sample-dependent data bias

To test for biases in the sequencing, we compared the sequences generated from three tiger reserves: (a) Kanha Tiger Reserve, (b) Wayanad Wildlife Sanctuary, and (c) Ranthambore Tiger Reserve in this study with those generated by Natesh et al., 2017, from the tissue samples, which were deposited as ddRAD data. All reads were trimmed and aligned to the tiger genome as described previously. SNPs were called as described here previously.

If there were systematic holistic biases in the data from shed hair, we expected that they would form a cluster separate from the tissue sequences. To recover the population structure obtained by Natesh et al. (2017), we used the filtered SNP dataset. Structure was estimated using the program fastSTRUCTURE (Raj, Stephens, & Pritchard, 2014) for complexity values of 2, 3, 4, and 5. The complexity value with the maximum likelihood are shown.

#### 2.4.4 Matrilineage analysis

We estimated the matrilineage of the tigers in our dataset for which we sequenced whole genomes. We mapped whole genome sequencing reads to the Amur tiger mitogenome as a reference (RefSeq NC_010642.1). Duplicates were marked. We then called consensus mitogenomes using ANGSD (Korneliussen, Albrechtsen & Nielsen, 2014). We used a mapping quality filter of 30, a minimum base quality of 30, minimum depth of 20, and maximum depth of twice the average sequencing depth. The average sequencing depth varied from 900X to 25,000X. We removed sites from the analysis that had any missing data. To test that the mitogenomes are free of biases, we reconstructed a known mitochondrial network. We performed multiple sequence alignments (MSA) of whole mitogenomes from T16, T20, and T64 using clustal-omega (Sievers et al., 2011), and then, a minimum spanning network was created using popart (Leigh & Bryant, 2015). We also used DnaSP version 6 (Rozas et al., 2017) to create a list of haplotypes (Baltazar-Soares & Eizaguirre, 2016). This tested if we recover the known matrilineages. Then, we inferred previously unknown matrilineage for T24, T47, and T104 using the same approach. We expect a single mutation between individuals to arise spontaneously (Baltazar-Soares & Eizaguirre, 2016; Tsai, Rajasekar, & John, 2016) and hence ignore a single mutational distance for the scope of this study.

## 3 Results

**Figure 1:**
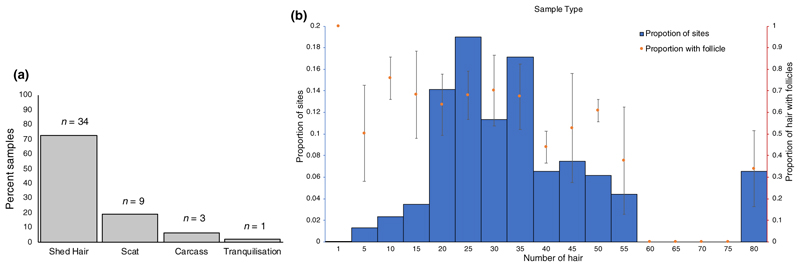
(a) In a period of 255 days of following 34 individuals, shed hair samples could be obtained for all 34 individuals while scat could be obtained for only 9. Opportunistically, 3 carcasses were recovered and 1 tranquilization was reported. (b) Number of hair strands collected per site. Out of the total hair strands collected per site, most sites seem to have at least 50% of the hair with follicle

We followed 34 individual wild tigers identified from their unique stripe patterns (Figure S1), and obtained shed hair samples from 207 sitting sites for these tigers. Ten scats samples were collected from nine of these individuals. Additionally, tissues from three opportunistically found tiger carcasses (death due to conflict) and one opportunistic tranquilization (Figure 1a) were obtained. From the 207 hair collection sites, we obtained on an average 25 hair strands per site (Figure 1b), of which approximately 65% strands had a potential hair root (Figure 1b).

**Figure 2:**
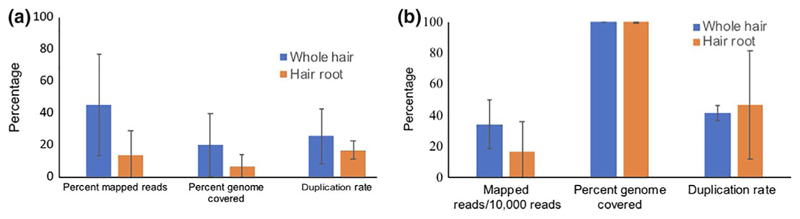
Whole hair DNA extracts have more reads mapping to the tiger reference nuclear genome and cover more bases on the nuclear genome (a) and also on the mitochondrial genome (b). The number of reads obtained from all sequences has been normalized

To evaluate the best strategy for DNA extraction and sequencing, shed hair from a wild-caught tiger T24 housed in zoo and a wild tiger T47 were used. Though the initial DNA concentration from the extracts was low, library prep and sequencing strategies did yield usable data. Sequencing of the DNA from hair root extracts yielded 13,452,410 and 373,791,866 reads while that from the whole hair yielded 15,735,782 and 341,232,300 reads (after adapter trimming) from T24 and T47, respectively. The DNA from the whole hair had higher percent mapped reads to nuclear and mitochondrial DNA of tiger and covered more of the genome compared to DNA from hair root only. The whole hair DNA extract had more tiger DNA and less bacterial DNA (Figure S2). However, the duplication rate for reads aligned to nuclear and mitochondrial genome (indicating PCR duplicates) was higher in whole hair DNA extracts (Figure 2a,b).

Across the five genome sequences of shed hair samples, the sequence quality in terms of percent mapped reads and percent genome covered was variable (Table S2). The minimum percent nuclear genome covered in our dataset was 24.85% (yielding 126,129 SNP loci) for shed hair from the tiger T20 and was maximally 98.03% (yielding 512,689 SNP loci) for tiger T47. Increasing the sequencing depth increased the percent genome covered.

**Figure 3:**
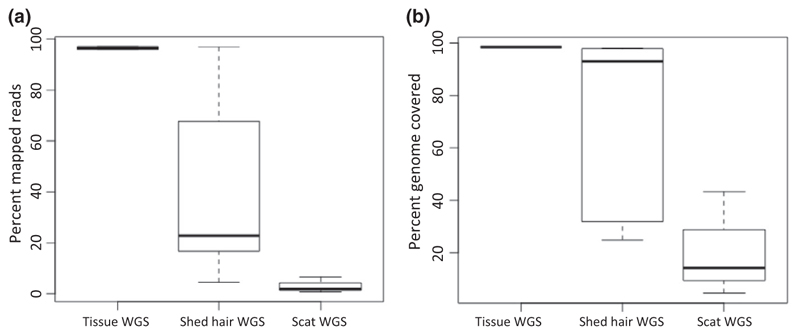
(a) Sequencing reads from tissue samples (*n* = 2) have higher mapped reads and low variance to the tiger nuclear genome as compared to hair (*n* = 5) or scat samples (*n* = 3). Hair samples have very high variance. (b) Tissue samples perform better in terms of percent genome covered

To test how shed hair performed in comparison to DNA from tissue and scat, we compared DNA sequences from tissues (whole genome sequencing (WGS) data of tigers T16 and T104), genome sequences from shed hair, and scat DNA genome sequences (tigers T03, T08 and T47). The number of nucleotides sampled was normalized across all samples. Tissue samples performed best, while scat samples performed the worst in terms of mapped reads, and percentage genome covered (Figure 3). The variance in the shed hair genome sequencing data was high, but overall, the average was better than scat.

### 3.1 Do genome-wide data from shed hair provide meaningful results?

**Figure 4:**
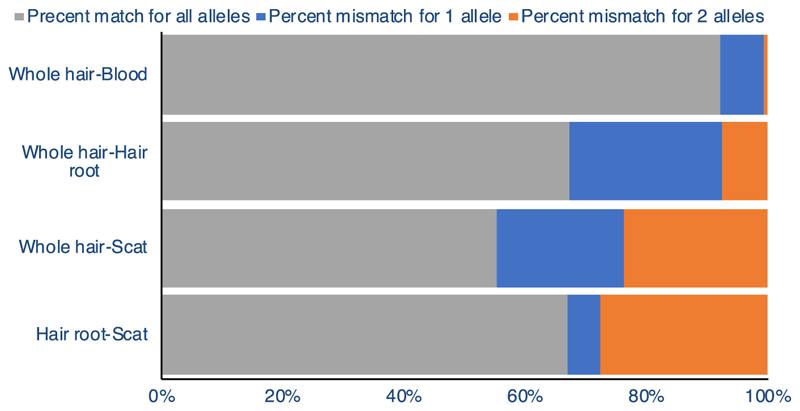
Percent pairwise mismatch between SNP data from different sample types of an individual

We compared mismatches between different sample sources. Comparison of whole hair and blood from tiger T104 showed that 92% of loci had zero mismatches while 7.4% of the loci had a single mismatch and 0.6% mismatched for both the alleles (Figure 4). Scat versus hair root and scat versus whole hair from tiger T47 had 67% and 55% of the loci without mismatches, while 5.3% and 21.2% of the loci had 1 mismatch, respectively. Comparison between hair root and whole hair (for tiger T47) revealed that 67.3% of the loci had no mismatches while 25.2% of the loci mismatched for 1 allele. We obtained similar results with the unmasked genome (Figure S5).

**Figure 5:**
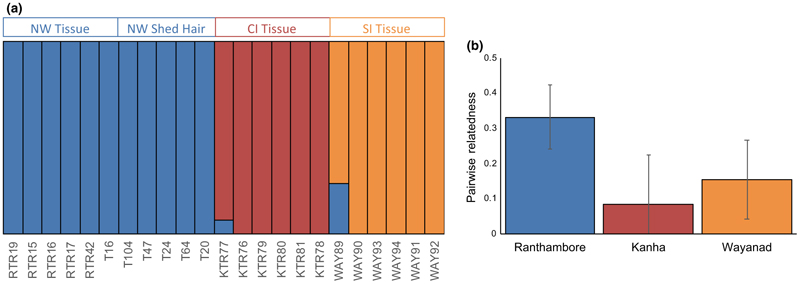
Results from Natesh et al. (2017) could be replicated after adding in the shed hair whole genome sequences. (a) If there were specific biases in the shed hair data, shed hair samples would have formed a separate cluster. The optimal complexity was 3. NW = Ranthambore Tiger Reserve, CI = Kanha Tiger Reserve and SI = Wayanad Wildlife Sanctuary. (b) The trends in pairwise relatedness for tiger reserve are similar in our dataset and that in Natesh et al. (2017). Ranthambore has the highest pairwise relatedness among the tiger reserves here consistent with Natesh et al. (2017)

We combined our data with those from three tiger populations sampled in Natesh et al. (2017): Kanha Tiger Reserve, Wayanad Wildlife Sanctuary, and Ranthambore Tiger Reserve. After filtering, we had 15,644 SNPs from this combined dataset. Results from *fastStructure* replicated the optimum complexity of 3 (Figure 5a). Results from higher complexity are presented in Figure S4 and reiterate this. We did not find any grouping (between sample types or otherwise) within Ranthambore (Figure 3). The relatedness estimates also revealed patterns similar to that in Natesh et al. (2017), with Ranthambore having the highest average pairwise relatedness (Figure 5b).

The tiger population in Ranthambore Tiger Reserve has been monitored closely by the forest department staff since its inception by daily observations, occasional tracking of individuals, and camera trapping. Due to their efforts, maternity and sib-ship relationships are known for several tigers. From this, the matrilineage of tigress T16 is thought to be one of the founders, and most tigers are supposed to have descended from her, making the relatedness between individuals high. However, certain tigers have no known maternity or matrilineage, thus demanding investigation. The tigers T20 and T64 are supposed to share T16’s matrilineage. While the mother of T24 is thought to be T22 and that of T104 is thought to be T41. T47’s maternity and matrilineage both are unknown.

**Figure 6:**
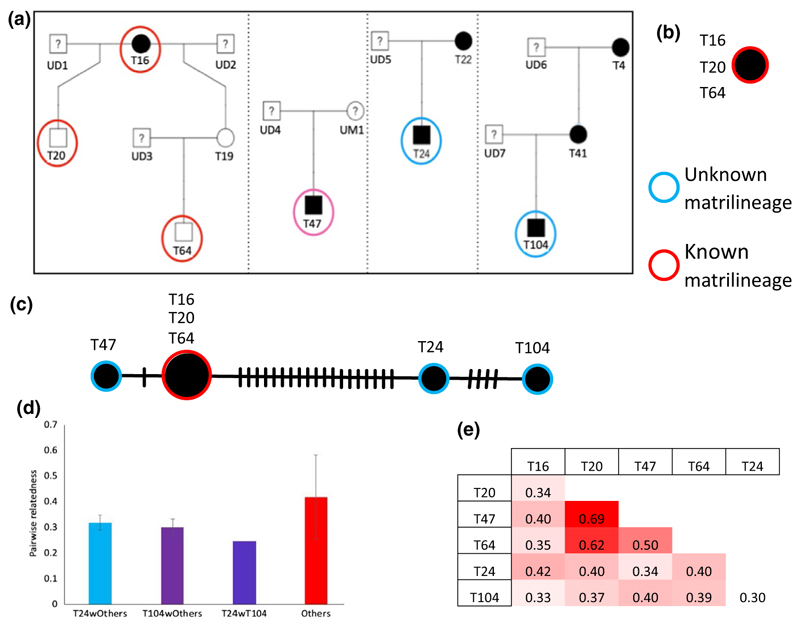
(a) The estimated pedigree of Ranthambore Tiger Reserve tigers from behavioral observations. The individuals in circles are sampled here and the individuals in red circles have known ancestry. The ticks on the lines represent a substitution between the nodes. (b) The estimated minimum spanning network for mitochondrial genome of T16, T20, and T64 with known ancestry. This is consistent with the behavioral data. (c) The estimated minimum spanning network for mitochondrial genome for all individuals in our dataset. (d, e) The pairwise relatedness using nuclear SNPs. “Others” are pairs of samples that exclude T24 and T104

As expected from the long-term data depicted in Figure 6a, we find T16, T20, and T64 belong to the same lineage (Figure 6b). Thus, we recovered known matrilineage reliably. This indicates that contaminations from nontiger DNA do not affect the mitogenome data. Using data from all 6 individual’s genomes analyzed here, we obtained the haplotype network depicted in Figure 6b. The network suggests that T47 belongs to same matrilineage as T16, T20, and T64 while T24 and T104 potentially belong to a different matrilineage. Additionally, pairwise relatedness (using 15,644 SNPs from the nuclear genome) between T24 with others and T104 with others is lower than pairwise relatedness estimates between half/full siblings T20, T47, and T64. The maximum relatedness of T24 is to T16 at 0.35 while the minimum relatedness is of T104 with T24 at 0.25 and the maximum is for T20 -T47 pair at 0.69 (Figure 6c,d). Thus, T47 might be a previously unknown full sibling of T20 and both sons of T16.

## 4 Discussion

### 4.1 Sample collection

Our results suggest that shed hair from identified individuals is an adequately available and effective source of DNA for generating genome-wide data and estimating relatedness with a potential for recovering pedigrees. Thus far, such individual-based molecular studies have been conducted mostly with captured and tagged individuals involving invasive sampling (e.g., Soay sheep, red deer (Clutton-Brock & Pemberton, 2004), meerkats (Leclaire, Nielsen, Sharp, & Clutton-Brock, 2013; Ross-Gillespie & Griffin, 2007) and Wolves (Vonholdt et al., 2008)) or with baited hair traps (e.g., red fox, Vine et al., 2009; black bear, Gardner, Royle, Wegan, Rainbolt, & Curtis, 2010; marten, Mowat & Paetkau, 2002; Eurasian lynx, Davoli, Schmidt, Kowalczyk, & Randi, 2013; Southern hairy-nosed wombats, Walker, Sunnucks, & Taylor, 2008 and Ocelots, Weaver, Wood, Paetkau, & Laack, 2005). Our results reveal that shed hair is a viable sample source for individual-level genetic studies. While shed hair sampling has been used (e.g., captive Panda, *Ailuropoda melanoleuca* (Durnin, Palsbøll, Ryder, & McCullough, 2007) and wild Orang-Utans, *Pongo pygmaeus*, (Goossens, Abdullah, & Sinyor, 2004)), in population genetic studies, we are not aware of any studies that have used whole genome sequencing methods. We show that shed hair is a viable source of noninvasive DNA and within this tiger population was a more abundant source compared to scat or carcasses. We suggest that collection of shed hair may allow individual and population-level whole genome-based studies in a relatively short span of time. This is especially important for conservation biology studies as scientifically informed decisions are often delayed due to difficulties in collecting samples from identified individuals.

Individual-level genetic studies become important especially when genotype-phenotype associations are of interest. Such studies are rare for populations of wild carnivores. Given of ongoing habitat fragmentation, population isolation, and climate change, it might be important for conservationists to understand whether a trait is heritable or driven primarily by the environment before implementing genetic rescue for the trait. However, for such studies one needs to sample individuals such that a genotype can be associated with the phenotype of an individual. In such cases, collection of identified samples becomes very important.

The population described here is one of the few high density tiger populations (e.g., Karanth, Nichols, Kumar, Link, & Hines, 2004). This high density contributes to the sampling rates we report here. For populations with low densities or difficult terrain, baited hair traps in conjunction with camera traps can be used to collect samples, especially in the case of species without pelage patterns. Shed hair samples have also been collected from nests of apes (Goossens et al., 2004). Similarly, individual-level sampling rates are also expected to be variable, and in some cases, baiting may help. However, baiting for hair traps is not allowed in several areas and hence our sampling strategy might be of help. Methods described here also benefit studies involving census and monitoring of populations where associating samples to a known individual may not be important. Additionally, individuals can also be tracked genetically using shed hair. This can also help in creating a genetic repository for a population for forensics or for mitigating conflicts.

The sampling strategy described here can potentially lead to collection of hair strands from multiple individuals leading to contamination. To avoid this, we do not sample from areas like water bodies or individuals with young cubs that remain physically very close to their mothers. We do not sample from mating or fighting sites either. Additionally, sampling the same individual multiple times and genotyping each sample with an SNP panel (Natesh et al., 2019) to establish consistency before whole genome sequencing.

#### 4.1.1 DNA sequencing and data quality

We observed that the initial DNA quantity from whole shed hair extracts was low but could be used for sequencing. We observed variance in the data quality obtained from the samples in terms of the percent host DNA, duplication rate, and percent genome covered. These measures although poorer than those from tissue samples do not affect our ability to detect population structure and patterns of average relatedness between individuals in a populations. The percent mismatch between SNPs from shed hair and tissue was only 2% and mismatched only for 1 of 2 alleles. This however needs to be tested more rigorously by increasing the number of such comparisons. The matrilineages detected from mitochondrial genomes also seem to be free of biases since they traced known matrilineages exactly (Figure 6b). From these observations, it seems that the difference in data between shed hair DNA and tissue DNA sequence might arise from the starting DNA concentrations. The starting DNA concentrations can be improved by using more efficient DNA extraction methods.

Ecological and evolutionary genetics studies can benefit greatly from advances in next-generation sequencing methods. However, obtaining good samples for wild individuals has always been a challenge. Using noninvasive samples can be an alternative. Methods that allow noninvasive samples to be used for obtaining genomic scale data like host DNA enrichment in scat samples (Chiou & Bergey, 2018), salivary samples from predatory bite marks (Blejwas, Williams, Shin, McCullough, & Jaeger, 2006), or baited camera traps (Shardlow & Hyatt, 2013) are possibilities. For optimal use of shed hairs, better methods of DNA extraction are needed. The host DNA yield from 10 to 12 shed hair is often low and cannot be quantified with straightforward methods such as qubit fluorometers. Hair metagenome is known to have several nonhost (contaminating) DNA fragments and more so in the hair roots owing to its relatively porous nature (Miller et al., 2008). Methods that can increase the efficiency of lysis in conjunction with enrichment methods will reduce contamination, thus increasing the overall host DNA content. This will have a twofold advantage of reducing potential sources of bias during the analysis and yielding more usable sequence data per unit raw data. The Chelex extraction method used by Bjornerfeldt and Vilà (2007) used to obtain DNA from single hair needs to be tested on shed hair too but it would be significantly more expensive than the method described here. Advances in low DNA concentration library preparation method followed by short-read sequencing will enable workers to use noninvasive samples more effectively. It is possible to use the method presented here to develop a genome-wide SNP panel for a species. Such a panel can then be used in conjunction with chelex-based extraction method followed by low DNA concentration library preparation to obtain data from single hair strands. Whole genome sequences from noninvasive samples will help in accurate and faster studies quantifying inbreeding using runs of homozygosity, identifying adaptive or deleterious alleles, and identifying functional genomic regions for endangered charismatic mammals.

#### 4.1.2 Matrilineages and relatedness in Ranthambore Tiger Reserve

Our results point to at least two new matrilines in Ranthambore Tiger Reserve (RTR). This population has undergone several bottlenecks, the most recent one in the year 2005, with few founders including T16. Bottlenecks are known to reduce allelic diversity, and hence, one might expect lower numbers of mitochondrial haplotypes in RTR compared to other populations. Singh, Qureshi, Sankar, Krausman, and Goyal (2013) have studied the dispersal of tigers in the landscape, and no evidence has so far been presented on immigration of tigers into RTR. In such a scenario, discovering a previously unknown matrilineage of tigers in RTR suggests the potential for additional founder lineages or potentially undocumented immigration. The matrilineage of tiger T24 was inherited from his mother T22 whose presence was detected as a adult in 2006 (Sadhu et al., 2017) and its whereabouts before this are unknown. Similar is the case for tiger T4 who is the grandmother of T104. Such lineages can supplement the population with genetic variation. Recovering matrilineages can prove to be important when estimating the pedigree of a population using several SNP markers from the nuclear genome. The matrilineages can be used as priors for the estimation of pedigrees. They can also be used for annotating the pedigree recovered from SNP markers. Thus, discovering matrilineages undocumented in observation-based data is important for recovering a wild pedigree.

Although we added samples to the Ranthambore dataset from Natesh et al. (2017) the pairwise relatedness between individuals in this population based on several thousands of SNPs remains high. This suggests the possibility of inbreeding in this population and that most individuals are highly related. Actual inbreeding can be tested using methods to estimate runs of homozygosity, based on sampling and genome sequencing from several individuals as described here. Inbreeding and inbreeding depression (if any) needs to be estimated and incorporated into management plans for this and other such isolated populations. The same sampling strategy could allow us to estimate the pedigree using genotypes of several individuals, and correlates of inbreeding depression by estimating differences in the number of successful offspring between inbred and outbred individuals. Such studies will allow us to investigate inbreeding avoidance, heritability of traits, heritability of territories, correlation of life history traits and genotype and several other traits for tigers.

Whole genome sequences have helped in discovering disease-causing and protective alleles (Epstein et al., 2016; Murchison et al., 2012), estimating inbreeding (Kardos et al., 2018) and low genetic variation (Abascal et al., 2016), planning and measuring success of genetic rescues (Saremi et al., 2018), estimating demographic history (Palkopoulou et al., 2015; Xue et al., 2015), ascertaining management units (Liu et al., 2018), and development of tools for several applications including monitoring populations (Faivre-Rampant et al., 2016). Having noninvasive samples that can be used for whole genome sequencing will be an advantage for endangered species research as it may lead faster turnaround time.

## 5 Conclusions

We aimed to identify the best noninvasive sample types for studying genetics of identified individuals and test if such samples can actually be used for genetic studies. We find that shed hair samples from identified individuals are the most frequently available sample types and DNA sequences from whole shed hair are better than using only the hair roots. We establish that the sequences obtained from whole hair are reliable and match 96% of the sequence obtained from blood. However, we do find large variations in the amount of data obtained from whole shed hair and that the DNA obtained is generally of low concentration. In the future, it might be possible to also use probe-based approaches to extract information on specific loci and/or genomic regions to enable most appropriate use of shed hair samples. In summary, we suggest that shed hair is a viable source of genome-wide data at the individual level from a wild population.

## Supplementary Material

Supplementary Material

## Data Availability

All sequences have been deposited in the SRA database with accession number PRJNA559670.
