# Supplementary material for: Are shed hair genomes the most effective noninvasive resource for estimating relationships in the wild?
Source: Ecol Evol. Author manuscript; Available in PMC 2020 Jun 17. (PMC7297754; doi:10.1002/ece3.6157)
Supplement: Supplementary Material [file EMS204995-supplement-Supplementary_Material.docx]

**Appendices**

**Table S1:** The samples used in present study

| Individuals | Samples collected | DNA sequence source |
| --- | --- | --- |
| T16 | Muscle Tissue from corpse | Tissue |
| T20 | Shed hair | Whole Hair |
| T64 | Shed hair | Whole Hair |
| T24 | Shed hair | Whole Hair |
|  |  | Hair Root |
| T47 | Shed hair | Whole Hair |
|  |  | Hair Root |
|  | Scat | Fecal |
| T03 | Scat | Fecal |
| T08 | Scat | Fecal |
| T104 | Shed hair | Whole Hair |
|  | Blood from tranquilization | Tissue |

**Table S2:** Sample sequencing data quality

| Individual | DNA source | Percent mapped reads | Percent genome covered | Total number of reads |
| --- | --- | --- | --- | --- |
| T104 | Whole hair | 96.91 | 97.91 | 332,479,846 |
| T20 | Whole hair | 4.51 | 24.85 | 162,272,498 |
| T64 | Whole hair | 16.79 | 31.89 | 343,777,966 |
| T47 | Whole hair | 67.68 | 98.03 | 341,232,300 |
| T24 | Whole hair | 22.87 | 93.04 | 393,167,528 |
| T47 | Scat | 6.63 | 43.32 | 185,253,494 |
| T03 | Scat | 1.94 | 14.2 | 177,066,138 |
| T8 | Scat | 0.77 | 4.56 | 132,602,774 |
| T104 | Tissue | 97.05 | 98.49 | 304,471,110 |
| T16 | Tissue | 95.89 | 98.49 | 710,167,210 |

**Table S3:** SNP filters and individuals used in specific analysis

| Type of Analysis with DNA sequence data | Tool | VCF filters | Samples used | Number of SNPs |
| --- | --- | --- | --- | --- |
| Percent Mismatch of scat with whole hair | PLINK | MinQ 30, minGQ 30, minDP 10, mm 0, rmvIndels | T47 WH, T47 SC | 1,213,803 |
| Percent Mismatch of scat with hair root | PLINK | MinQ 30, minGQ 30, minDP 10, mm 0, rmvIndels | T47 SC, T47 HR | 1,213,803 |
| Percent mismatch of whole hair with hair root | PLINK | MinQ 30, minGQ 30, minDP 10, mm 0, rmvIndels | T47 WH, T47 HR | 2,917,519 |
| Percent mismatch of whole hair with tissue | PLINK | MinQ 30, minGQ 30, minDP 10, mm 0, rmvIndels | T104 WH, T104 TS | 4,353,417 |
| Structure between whole hair and tissue | fastSTRUCTURE | MinQ 30, minGQ 30, minDP 10, mm 0.8, hwe 0.05, mac 3, rmvIndels | Data from Natesh et al 2017, T16 TS, T104 WH, T47 WH, T64 WH, T20 WH, T24 WH | 15,644 |
| Within Ranthambore structure between samples | fastSTRUCTURE | MinQ 30, minGQ 30, minDP 10, mm 0.8, hwe 0.05, mac 3, rmvIndels | RTR Data from Natesh et al 2017, T16 TS, T104 WH, T47 WH, T64 WH, T20 WH, T24 WH | 15,645 |
| Pairwise relatedness | PLINK | MinQ 30, minGQ 30, minDP 10, mm 0.8, hwe 0.05, mac 3, rmvIndels | Data from Natesh et al 2017, T16 TS, T104 WH, T47 WH, T64 WH, T20 WH, T24 WH | 15,646 |

**Appendix Method:** Assessing read content in the samples

The bioinformatics pipeline used is depicted in Supplementary Box 1.

Read quality check **-** We check few parameters from fastq file such as i) Base quality score distribution, ii) Sequence quality score distribution, iii) Average base content per read, iv) GC distribution in the reads, v) PCR amplification issue, vi) Check for over-represented sequences, vii) Adapter trimming. Based on quality report of fastq files we trim sequence read where necessary to only retain high quality sequence for further analysis. In addition, the low-quality sequence reads are excluded from the analysis. The adapter trimming was performed using Trimmomatic-0.36.

Read alignment – The paired-end reads are aligned to the Panthera tigris reference genome downloaded from NCBI database. Genome fasta sequence was received from website (https://www.ncbi.nlm.nih.gov/genome/?term=PRJNA182708). Alignment was performed using Bowtie2 (fast and sensitive read aligner) program (version 2.2.1).

Read alignment to human - The unaligned paired-end reads from above process is mapped to human reference genome Feb. 2009 release downloaded from UCSC database (GRCh37/hg19). The chromosome fasta file was downloaded from the following website

http://hgdownload.soe.ucsc.edu/goldenPath/hg19/bigZips/chromFa.tar.gz

Read alignment to Mouse - The unaligned paired-end reads from above process are aligned to the reference Mouse genome GRCm38 downloaded from Ensembl database.

ftp://ftp.ensembl.org/pub/release-78/fasta/mus_musculus/dna/Mus_musculus.GRCm38.dna.toplevel.fa.gz

Read alignment to Bacteria - The unaligned paired-end reads from above step are aligned to a reference database made from all the reference bacterial genomes downloaded from NCBI database.

Alignment with all four reference genomes was performed using Bowtie2 (fast and sensitive read aligner) program (version 2.2.1). We also tried changing the order of alignment to reference genomes.

**a)**

**b)**

**Box S1**: Bioinformatics pipeline for assigning read identities a) consecutive mapping b) competitive mapping


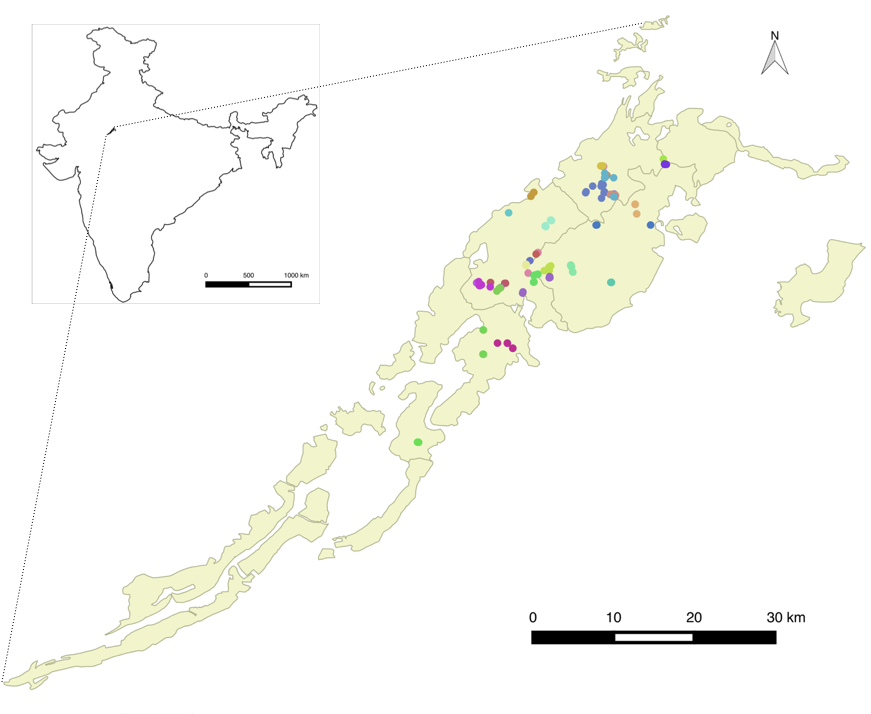


**Figure S1:** Study location. Ranthambore Tiger Reserve is in north-western India. Every dot on the map represents a sampling location and different colours represent different individuals. A total of 38 individuals were sampled.

**a)**

**b)**

**Figure S2:** Percent read content in individual samples using a)Consecutive mapping. Changing the order of alignment to reference genome does not change the results significantly. (b) Using competitive mapping.

K=4

K=3

K=2

K=5

**Figure S3:** To test if biases were subtle enough to be detected at an intra-population scale structure was estimated using the shed hair and the Ranthambore samples in Natesh et al (2017). However, no structure is detected thus there are biases to create population level errors. At high K values the percent ancestry of other cohorts is very low and hence not visible.

K=4

K=5

**Figure S4:** Results from Natesh et al. (2017) could be replicated after adding in the shed hair whole genome sequences. If there were specific biases shed hair WGS samples would have formed a separate cluster. The optimal complexity was 3. The hair samples do not form a separate cluster at higher K values. NW= Ranthambore Tiger Reserve, CI= Kanha Tiger Reserve and SI= Wayanad Wildlife Sanctuary. At high K values the percent ancestry of other cohorts is very low and hence not visible.

**Figure S5:** Percent pairwise mismatch between SNP data from different sample types of an individual using the unmasked genome as reference.

**Reference**

Korneliussen, T. S., Albrechtsen, A., & Nielsen, R. (2014). ANGSD: analysis of next generation sequencing data. *BMC bioinformatics*, *15*(1), 356.
